# *In vivo* genotoxicity of Chios mastic gum in rodent bone marrow micronucleus test

**DOI:** 10.1016/j.toxrep.2025.102155

**Published:** 2025-10-31

**Authors:** Eirini-Christina Psarou, Aikaterini Termentzi, Katerina Kyriakopoulou, Pelagia Anastasiadou, Marios Meidanis, Nikolas Fokialakis, Kyriaki Machera

**Affiliations:** a Laboratory of Toxicological Assessment of Pesticides, Benaki Phytopathological Institute, 8 Stefanou Delta Street, Kifissia , Athens 14561, Greece; b Division of Pharmacognosy and Chemistry of Natural Products, Department of Pharmacy, National and Kapodistrian University of Athens, Athens 15771, Greece

**Keywords:** Chios mastic gum, Genotoxicity, Mammalian erythrocyte micronucleus test

## Abstract

Chios mastic gum (CMG) exhibits several pharmacological activities that have been confirmed by numerous studies. These include antibacterial, anti-inflammatory, hypolipidemic, hypoglycemic, antiatheromatic, and benefits for against gastrointestinal disorders. However, studies focusing on CMG’s safety are limited. The aim of the present study is to evaluate the genotoxicity of CMG using the limit test on the mammalian erythrocyte micronucleus assay, in male Wistar rats. CMG was administered by gavage to 5 rats at 2000 mg/kg bw for 3 days, while 5 rats received the vehicle and 5 rats received cyclophosphamide (positive control). Satellite groups of 3 rats were included for the negative control and CMG-treated groups to collect plasma and bone marrow for the chemical analyses. All rats were observed for mortality and clinical signs of toxicity during the dosing period. Rats were euthanatized 20 h after the last treatment, necropsied, and bone marrow was collected for smear preparation. No mortality, clinical signs of toxicity, gross organ pathology, or body weight changes were observed in CMG-treated rats compared to the negative control group. No statistically significant alteration of polychromatic erythrocytes (PCE) and the per-thousand incidences of micronucleated PCE in the bone marrow of CMG-treated rats were observed. Bone marrow exposure to CMG was unambiguously confirmed by the detection of the characteristic CMG triterpenic acids in both bone marrow extract and plasma of CMG-treated rats by UHPLC-HRMS/MS analysis. In conclusion, CMG exhibited no genotoxic effects on bone marrow erythrocytes at the tested limit dose of 2000mg/kg body weight.

## Highlights

• **First** ***in vivo*** **genotoxicity study** of Chios mastic gum (CMG) using mammalian erythrocyte micronucleus assay in Wistar rats.
• **No evidence of genotoxicity**: no increase in micronucleated polychromatic erythrocytes (PCEs) compared to control.
• **No toxicity observed**: no mortality, clinical signs, gross organ pathology, or body weight changes in CMG-treated animals.
• **Exposure confirmation**: detection of characteristic CMG triterpenic acids in both plasma and bone marrow by UHPLC-HRMS/MS.
• **Robust negative result** strengthens the safety profile of CMG, supporting its use in nutraceuticals, food, cosmetics, etc.

## Graphical Abstract

**Figure:**
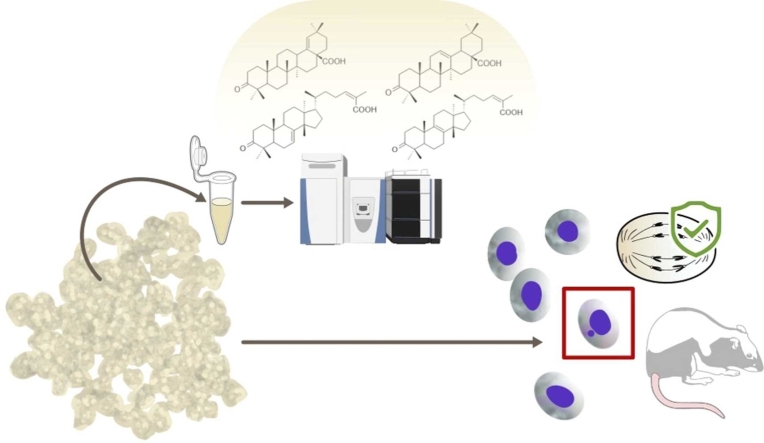

## 1 Introduction

Chios mastic gum (CMG), known also as Chios Mastiha, is the resin of the plant *Pistacia lentiscus* L.*,* which is exclusively cultivated in Chios Island, Greece. CMG has been traditionally used in Greek medicine to treat various disorders since antiquity [58]. Recently, it has been recognized as a traditional herbal medicine by the European Medicines Agency (EMA) for indications including mild dyspeptic disorders, skin inflammation and wound healing ([16], [17], [18]).

Expansive studies have supported additional therapeutic properties of CMG. These include antibacterial [44], [57], anti-inflammatory [28], [29], [42], [63], [69], hypolipidemic [19], [31], [5], [61], [63], hypoglycemic [19], [31], [63] and antiatheromatic [11], [5], as well as activity against gastrointestinal disorders [2], [28], [29], [3], [44], [57]. Given the growing scientific evidence, CMG is emerging as a promising therapeutic and protective natural product against multiple disorders, such as diabetes [19], [63], cardiovascular disorders [5], [61] and gastrointestinal diseases such as Chrohn’s disease (CD) and Irritable Bowel Syndrome (IBS) [10], [20], [28], [29].

Phytochemically, CMG gum is a complex natural product comprising over 120 characterized chemical compounds [53] that can be divided into three major categories involving a) the polymer, b) the volatile fraction (essential oil) and c) the triterpenic fraction [53]. The polymer of CMG, which comprises approximately 25 % of the resin weight, is consisted of poly-β-myrcene [65]. The volatile fraction, approximately 2–3 % of the resin’s weight, is mainly consisted of monoterpenic hydrocarbons, oxygenated monoterpenes and sesquiterpenes [36], [43], [56]. The triterpenic fraction is the major chemical category of CMG, comprising 65–70 % of the resin weight, and it mainly contains tetracyclic and pentacyclic triterpenes. The triterpenic fraction can be further chemically separated into acidic and neutral parts. The major constituents of the acidic fraction are oleanonic acid, moronic acid, iso/masticadienonic acids and iso/masticadienolic acids, while the neutral fraction consists mainly of triterpenic alcohols and aldehydes [55], [57], [6], [68]. Pharmacological properties of CMG are largely attributed to the triterpenic constituents of CMG [21], [31], [42], [5], [54], [57], [59], [64].

For herbal substances and preparations, preclinical toxicity testing is not required for their approval and registration as traditional herbal medicinal products, since their safety is demonstrated by their documented long-term medicinal use according to relevant scientific literature on safety data [14]. On the other hand, [15] document reports the need of genotoxicity testing for pharmaceuticals intended for human use. Specific genotoxicity, and generally adequate toxicity studies have not been performed for CMG ( [16], [17], [18]). In Mastic’s monograph and supporting documents from EMA is mentioned the lack of specific data on the mutagenicity of CMG ( [16], [17]). Vlastos et al. [67], [66] have only tested CMG water and oil by using the somatic mutation and recombination test in *Drosophila melanogaster*. This test indicated lack of genotoxic activity of the tested substances. Vlastos et al. [67], [66] similarly evaluated the genotoxic activity of CMG water and oil by the *in vitro* micronucleus test on human lymphocytes, indicating also lack of induction of micronuclei in lymphocytes at the concentrations tested. However, the *in vitro* studies do not provide any information related to the potential effects in an intact mammalian organism and the potential differences in the genotoxic potential of metabolites. Given that CMG is now recognized as a promising wide-range potential pharmaceutical agent for various disorders [53], [58], with increasing interest in the global nutraceutical and pharmaceutical market, the further investigation of its toxicity would be essential.

The testing of compounds for genotoxic activity provides information for hazard identification and more specifically, it is a potential indicator of carcinogenicity [26], [9]. *In vitro* genotoxicity tests are mainly used for screening purposes providing indications for further in vivo testing. However, *in vitro* results should be verified by *in vivo* assays, providing reliable conclusions [30], [62]. Since no single test system can detect all genotoxic mechanisms, a battery approach is generally used and recommended in the genotoxicity assessment of chemicals [7], [9]. Recommended test batteries for most regulatory authorities generally include tests that detect gene mutations, structural and numerical chromosomal damage in both *in vitro* and *in vivo* tests ( [47], [48], [49], [51], [13]). Although minor differences may exist, the genotoxicity testing strategy for most regulatory authorities is based on a battery of tests including a bacterial gene mutation assay, an *in vitro* mammalian cell assay for gene mutation and/or chromosome aberration detection and an *in vivo* assay for assessing chromosomal effects [7], [9].

Mammalian erythrocyte micronucleus test is one of the basic *in vivo* tests included in the battery of genotoxicity tests required by regulatory agencies globally for chemical safety assessment [37]. The assay is based on the detection of chemicals that cause chromosomal damage or mitotic apparatus dysfunction in progenitor red blood cells, resulting in the formation of micronuclei in the newly produced erythrocytes, the polychromatic erythrocytes (PCE) sampled either in the bone marrow or peripheral blood of animals [50]. An increase in the incidence of micronucleated PCEs in treated animals is suggestive of induced chromosomal damage caused by the treatment, which can be structural and/or numerical chromosomal damage. The assay is especially relevant for evaluating genotoxicity hazard, as it assesses cytogenetic damage of a chemical *in vivo*, taking into account factors such as metabolism, pharmacokinetics and DNA repair processes [50].

The present study constitutes the first *in vivo* genotoxicity evaluation of CMG employing the mammalian erythrocyte micronucleus test in male Wistar rats. Exposure of bone marrow to CMG was confirmed by the detection of its characteristic triterpenic acids in plasma and bone marrow of CMG treated rats using ultra-high performance liquid chromatography coupled with high-resolution mass spectrometry (UHPLC-HRMS/MS).

## 2 Materials and methods

### 2.1 Test substances and chemicals and materials

CMG was supplied by Chios Mastiha Growers Association (in the island of Chios, Greece), which is the exclusive worldwide producer of the resin. The dried extract of the acidic fraction of mastic (containing the characteristic triterpenic acids of CMG), which was subjected to UHPLC-HRMS/MS analysis, was provided by the Laboratory of Pharmacognosy and Natural Products Chemistry (National and Kapodistrian University of Athens) and has been isolated as described previously [68]. The acidic fraction was used to establish the UHPLC-HRMS/MS method and the identification of the characteristic mastic triterpenic acids. LC-MS grade methanol and formic acid (FA) were purchased from Merck (Darmstadt, Germany). Carboxymethyl cellulose and NaCl were purchased from Sigma-Aldrich. Phosphate-buffered solution, xylene, and microscopy slides were obtained from Fisher Scientific, while ethanol from Agros Chemicals. Blood collection tubes containing K_3_-EDTA were purchased from FL Medical. FBS was supplied by Life Technologies. Cyclophosphamide, Giemsa and May Grunwald stain solutions, Entellan were provided by Sigma-Aldrich.

### 2.2 Animal selection and housing conditions

Since the micronucleus formation is similar between male and female animals and most studies can be performed in either sex [50], the present study was performed on male rats. A total of 21 healthy and young adult male Wistar rats from the breeding colony of Benaki Phytopathological Institute were used (Registration codes EL25BIO027 and EL25BIO026). Ten-week-old rats at the start of the dosing collected from cages with different generators were randomly assigned to control and treatment groups, individually identified, and housed in polypropylene solid-floor cages. Weight variation among animals was maintained within ±20 % of the mean (278.6 ± 20.89 g) during allocation. All animals were kept under controlled environmental conditions (temperature 22 ± 2 ºC, relative humidity 55 ± 10 %, light: dark circle 12:12 h) with ad libitum access to certified laboratory feed (4RF25, Mucedola, Italy) and water. The rats were allowed acclimatized for five days before treatment initiation. All animal procedures complied with the current European and National Legislation [EU Directive on the protection of animals used for scientific purposes (2010/63/EU) and the Hellenic Presidential Decree No 56/2013]. The study protocol was approved by the Veterinary Department of the Prefecture of Attica (Ethical Committee approval No. 1611/11.04.2018).

### 2.3 Study design and sample collection

Genotoxicity testing of CMG was performed according to the principles of OECD 474 guideline for the mammalian erythrocyte micronucleus test [50]. The limit test for the mammalian erythrocyte micronucleus test was performed, based on previous studies in our lab [32], indicating the absence of any toxic effects of CMG extract in male Wistar rats at 2000 mg/kg bw dosing and the lack of genotoxic activity of CMG fractions in *in vitro* and *in vivo* tests [66], [67]. For this purpose, 5 rats were allocated in the CMG-treated group, while 10 rats were equally allocated in the negative and positive control groups. The number of animals used was in line with the principles of OECD 474 guideline for the mammalian erythrocyte micronucleus test [50]. The treated group received three consecutive daily doses of 2000 mg/kg bw CMG via oral gavage at a volume of 1 ml/ 100 g of body weight. Mastic suspension (CMG) was prepared in 2 % aqueous carboxymethyl cellulose solution, shortly before each administration. The dosing was split into two daily treatments within an interval of 2 h, for the first two days of treatment. A single dose was administered on the third day after an overnight fast of the animals. Negative control rats received only the vehicle at the same dose and frequency as the treated animals. Positive control animals received only a single dose of the positive control compound, namely 20 mg/kg bw cyclophosphamide in 0.9 % NaCl by oral gavage at a volume of 0.5 ml/100 g b.w. without prior fasting. Two satellite groups (negative control and CMG-treated), consisting of 3 rats each, were included in the study for blood and bone marrow collection, to confirm the bioavailability of key CMG metabolites by UHPLC-HRMS/MS analysis. All animals had unlimited supply of laboratory diet and drinking water throughout the study. Individual weights of rats were recorded at the beginning and at the end of the study. During the dosing period all rats were observed twice a day for mortality and clinical signs of toxicity.

Twenty hours after the third treatment, rats of the CMG-treated and negative control group were euthanatized for bone marrow collection. Euthanasia was carried out by exposure to an overdose of CO_2_. Rats belonging to the positive control group were euthanatized 24 h after treatment for bone marrow collection. Rats of the satellite groups were subjected to blood withdrawal 1 h after the third daily treatment. Blood sampling was performed by orbital sinus bleeding, following a general CO_2_ anesthesia of the animals. 1 ml of blood was collected and the plasma (∼250 μl) was stored (-80°C) for chemical identification. Finally, rats from the satellite groups were sacrificed to collect bone marrow supernatant in PBS after centrifugation (x 500 g for 1 min) for HRMS/MS analysis.

### 2.4 Bone marrow smear preparation and staining

Immediately after the gross examination of the animals, bone marrow was collected from the rat femur in 1.5 ml ice-cold FBS and the cells were pelleted by centrifugation at x 500 g for 1 min. Two slide smears were prepared from each rat femur of each animal, and they were allowed to air-dry at room temperature for at least 24 h. Staining of the bone marrow smears was performed by using Giemsa and May Grunwald stain solutions following the standard approach, which are commonly used for the morphological and differentiation assessment of cells in bone marrow smears under microscopic analysis [60].

### 2.5 Bone marrow scoring and statistical analysis

Bone marrow cells were manually scored under a light microscope at 1000x magnification. The specimen evaluation was carried out under blinded conditions to prevent operator bias. The ratio of immature erythrocytes versus the total number of erythrocytes (mature and immature) was determined for each animal by counting a total of 500 erythrocytes. The incidence of micronucleated immature erythrocytes was determined by scoring 2000 immature erythrocytes for each animal. Micronuclei visualization was facilitated by the absence of a nucleus in erythrocytes, however micronucleus identification was only considered positive when the micronucleus was rounded-shaped, deeply stained, lied in the same focal plane as the cell, large enough to discern its morphological characteristics and when micronucleus-like debris were not present in the cell surrounding area [8]. After completion of the microscopic analysis, the percentage % of immature erythrocytes and the ‰ frequency of erythrocyte micronucleus in bone marrow of each experimental animal were calculated as a measure of cytotoxicity and genotoxicity of the test substance to erythroblasts respectively [37].

For statistical analysis both parametric and non-parametric tests are equally recommended for micronucleus data evaluation [34]. Data normality was assessed using the Shapiro-Wilk test for small size populations [45] to establish the more suitable statistical method. Results that passed normality test, were treated with one-way ANOVA analysis involving multiple comparisons with negative control group and multiple comparisons correction using statistical hypothesis testing by Sidak’s test. Results, which did not pass normality test, were curated with the non-parametric Kruskal-Wallis test involving multiple comparisons with negative control group and multiple comparisons correction using statistical hypothesis testing by Dunn’s test. All statistical analyses were conducted using GraphPad Prism 8.

### 2.6 UHPLC-HRMS/MS analysis of plasma, bone marrow and CMG extracts

Plasma and bone marrow samples were processed according to well established extraction protocols [41] with minor modifications. Briefly, plasma samples were thawed on ice, and 100 μL of each sample were collected and mixed with 300 μL iced-cold methanol. The sample was vortexed for 15 sec and centrifuged at 15800 g for 15 min at 4ºC. The supernatant was collected and 300 μL iced-cold methanol was added again to the pellet to repeat extraction. Samples were sonicated for 2 min, re-centrifuged and the resulting supernatants of each sample were pooled together. 500 μL supernatant of each sample were dried and concentrated by evaporation of the solvent with gas N_2_ stream for approximately 3 h, while samples were remaining in an ice bath. Dried samples were stored at −80 ºC until the UHPLC-HRMS/MS analysis of plasma. The day of the UHPLC-HRMS/MS plasma analysis 90 μL of prechilled MeOH:H_2_O (1:1) were added to each dried sample derived from plasma, the sample was vortexed for 15 s and centrifuged at 15800 g for 15 min at 4ºC. The final supernatant was transferred to screw-capped autosampler vial and stored in the autosampler of the UHPLC-HRMS/MS platform at 4ºC.

Bone marrow supernatants (500 μL) were thawed on ice and mixed with 1000 μL iced-cold methanol. The sample was vortexed for 15 sec and centrifuged at 15800 g for 15 min at 4ºC. The supernatant was collected and 1000 μL iced-cold methanol was added again to the pellet to repeat extraction. The resulting supernatants of each sample were pooled together in a microcentrifuge tube. 1000 μl supernatant of each sample were dried by evaporation of the solvent with gas N_2_ stream for approximately 4 h while samples remaining in an ice bath. Dry samples were placed at −80 ºC until the analysis. The day of the UHPLC-HRMS/MS analysis 300 μL of prechilled MeOH:H2O (1:1) were added to each dried sample, the sample was vortexed for 15 s and centrifuged at 15800 g for 15 min at 4ºC. The final supernatant was transferred to a screw-capped autosampler vial and stored in the autosampler of the UHPLC-HRMS/MS platform at 4ºC. All processes were temperature-controlled with samples remaining in an ice bath before and after the various treatments.

To confirm the presence of known compounds of CMG in plasma of CMG-treated rats, the dried extract of the acidic fraction of mastic was diluted in methanol: dH2O 1:1 at a concentration of 2 μg/ml and transferred to screw-capped autosampler vial for UHPLC-HRMS/MS analysis. The provided acidic fraction of mastic has been previously chromatographed in parallel with enriched standard compounds of CMG for phytochemical characterization of the main peaks of the fraction by LC-HRMS/MS [68].

UHPLC analyses were conducted on a Dionex Ultimate 3000 UHPLC system (Thermo Scientific, Germany) equipped with a binary pump, autosampler, online vacuum degasser, and temperature-controlled column compartment. A Hypersil Gold UPLC C18 (2.1 × 150 mm, 1.9 μm) reversed phased column (Thermo Scientific, Germany) was used for the analysis. The high-resolution mass spectrometry (HRMS) was performed on an Orbitrap Q-Exactive mass spectrometer (Thermo Scientific, Germany). Mobile phases consisted of 0.1 % (v/v) formic acid in water (mobile phase A) and 0.1 % (v/v) formic acid in methanol (mobile phase B). The mass spectrometric analysis was carried out in negative (ESI-) ion mode at a mass scan range of 100–1000 *m/z.* A 37 min gradient elution program was applied for plasma and mastic extract chromatographic analyses as follows: 0–15 m in. 95 % A: 5 % B, 15–30 m in. 30 % A: 70 % B, 30–35.1 min in 5 % A: 95 % B and 35.1–37 m in 95 % A: 5 % B. Flow rate was at 0.25 ml/min and column temperature was kept at 40 °C throughout the analyses. The injection volume was 2 μL for mastic extract and 3 μL for plasma samples. For the negative ion mode, the capillary temperature was set at 320° C, the sheath gas flow to 40 arb. units and the aux gas flow to 8 arb. units, the spray voltage to 2.7 kV and S-lense Rf level to 50 V. Analysis was carried out using the Fourier transform mass spectrometry (FTMS) mode in the full scan ion mode, having a resolution of 70,000. Mass spectra were acquired at profile mode applying a data dependent acquisition method, enabling MS/MS fragmentation of the three most intense ions. The MS/MS fragments were monitored at 35,000 resolving power.

## 3 Results

### 3.1 Animal live phase and autopsy

All rats (5/5) treated with CMG at 2000 mg/kg bw for three consecutive days survived until the end of the experiment when animals were euthanatized. All rats in the positive control group also survived until the end of the experiment. No clinical signs of toxicity were observed in any animals throughout the study period. Finally, results from autopsy examination did not show findings of gross organ pathology.

Body weights of CMG treated rats did not differ significantly from those of the negative (p = 0.3144) or positive control (p = 0.3736) groups at the beginning of the experiment, suggesting minimal weight variation of animals participating in the study (Table 1). Body weight of rats treated with CMG and positive control rats were not altered compared to the body weights of negative control group at the end of the experiment (p = 0.1394 and p = 0.2318 respectively), suggesting a lack of impact of treatment on animal body weights.

**Table 1:** Average body weights (g) of rats participated in the mammalian bone marrow micronucleus study at the start and the end of the experiment. The results are presented as mean (g) ± SEM. Statistical analysis was performed by one-way ANOVA involving multiple comparisons with negative control group in GraphPad Prism 8. *: p < 0.05, **: p < 0.01, ***: p < 0.001.

**Experimental group**	**Start**	**End**
**Negative control group**	266.6 ± 5.8	254.6 ± 5.4
**Mastic-treated group**	285.4 ± 11.1	277.2 ± 9.2
**Positive control group**	283.8 ± 7.3	273.6 ± 7.2

### 3.2 Bone marrow scoring

**Fig. 1:**
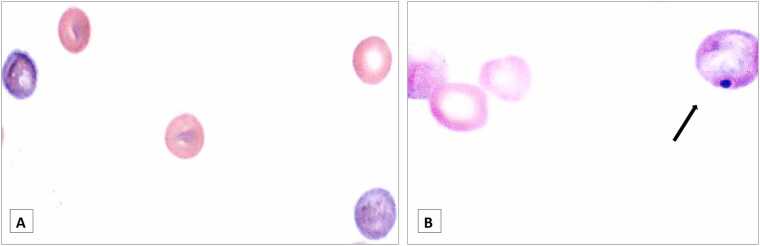
Representative pictures of bone marrow smears at 1000x magnification of (A) a negative and (B) a positive control rat in the mammalian erythrocyte micronucleus study. The arrow depicts the presence of a micronucleus in a polychromatic erythrocyte in the positive control sample (B).

Representative Giemsa and May Grunwald stained bone marrow smears under microscopic analysis are shown in Fig. 1. Immature erythrocytes (PCEs) containing larger residual amounts of RNA are stained more heavily with basophilic stains than do mature erythrocytes (NCE: normochromatic erythrocytes), resulting in a light blue or blue gray staining that differentiates them from mature erythrocytes which are stained orange-pink in conventional staining [37], [60], as it can be seen in Fig. 1A. An example of a micronucleus identification in a PCE cell from a positive control sample is shown in Fig. 1B.

Following microscopic analysis, individual and mean percentages of immature erythrocytes, PCE/NCE ratios and ‰ frequency of immature erythrocyte micronucleus (MNPCE/1000 PCE) were determined for each rat, as presented in Table 2.

**Table 2:** Individual and average percentages of immature erythrocytes (% PCE), immature/ mature erythrocytes ratios (PCE/NCE) and the frequencies of micronucleated immature erythrocytes (MNPCE/1000 PCE) in CMG-treated and control rats participated in the mammalian erythrocyte micronucleus study. The results are presented as mean ± SEM. Statistical analysis was performed by Kruskal-Wallis test involving multiple comparisons with negative control group in GraphPad Prism 8. *: p < 0.05, **: p < 0.01, ***: p < 0.001.

**Experimental group**	**Animal code**	**PCE/NCE**	**%PCE**	**MNPCE** **/1000 PCE**
**Negative control**	N1	0.83	45.33	3
	N2	0.79	44.05	3
	N3	0.78	43.93	4
	N4	1.02	50.38	4.5
	N5	0.98	49.52	3
Mean ± SEM		0.88 ± 0.05	46.64 ± 1.38	3.4 ± 0.4
**Mastic-treated**	M1	0.59	37.13	4.5
	M2	0.58	36.57	3.5
	M3	0.89	46.96	2.5
	M4	0.87	46.65	4.5
	M5	0.92	47.83	4
Mean ± SEM		0.77 ± 0.08	43.03 ± 2.53	3.8 ± 0.4
**Positive control**	P1	0.61	37.83	30.5
	P2	0.52	34.30	43
	P3	0.61	38.04	40
	P4	0.57	36.25	37
	P5	0.67	40.20	41
Mean ± SEM		0.60 *±0.02	37.33 *± 0.98	38.3 **± 2.2

Statistical analysis indicated that CMG treatment did not significantly affect the percentage % of immature erythrocytes (p = 0.7159), or the PCE/NCE ratio (p = 0.7151) in CMG-treated rats compared to negative control rats. In contrast, cyclophosphamide (20 mg/kg bw) treatment significantly reduced the percentage % of immature erythrocytes (p = 0.0267) and the PCE/NCE ratio (p = 0.0265) in positive control rats compared to negative control rats, suggesting a cytotoxic effect of that treatment on bone marrow erythropoiesis. Regarding the primary endpoint in the micronucleus assay, statistical analysis showed that CMG treatment did not increase the frequency of micronucleated immature erythrocytes (MNPCE) in the bone marrow smears of CMG-treated animals compared to negative control rats (adj.p > 0.9999), suggesting a lack of genotoxic effect of the test agent on erythroblasts. In contrast, cyclophosphamide treatment (20 mg/kg bw) significantly increased the frequency of MNPCE in the positive control group compared to negative control group (p = 0.0078), demonstrating the validity of the method, as it can be seen in Table 2.

### 3.3 UHPLC-HRMS/MS analysis of plasma and bone marrow extracts for mastic compounds identification

**Fig. 2:**
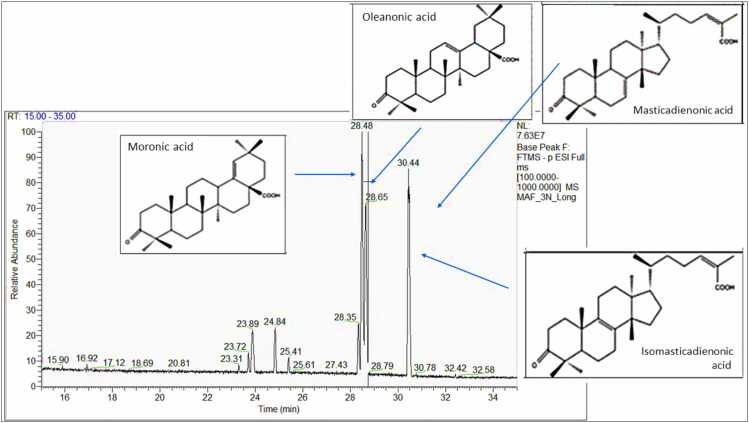
Base peak chromatogram of the acidic fraction from CMG extract after UPLC-HRMS/MS analysis in (-) ESI showing the three main peaks of the extract corresponding to moronic acid, oleanonic acid and iso/masticadienonic acid.

To confirm the presence of CMG compounds in plasma of treated rats, acidic fraction of CMG was analyzed by UHPLC-HRMS/MS alongside plasma and bone marrow samples. The base peak chromatogram of the acidic mastic fraction (Fig. 2) revealed three major peaks at retention times (Rts) of 28.48, 28.65 and 30.44. The three main peaks of the extract have been previously characterized by HRMS spectra and using enriched extracts in a similar chromatographic methodology. They correspond, in order of elution, to moronic acid, oleanonic acid and isomasticadienonic/masticadienonic acid respectively [68].

**Fig. 3:**
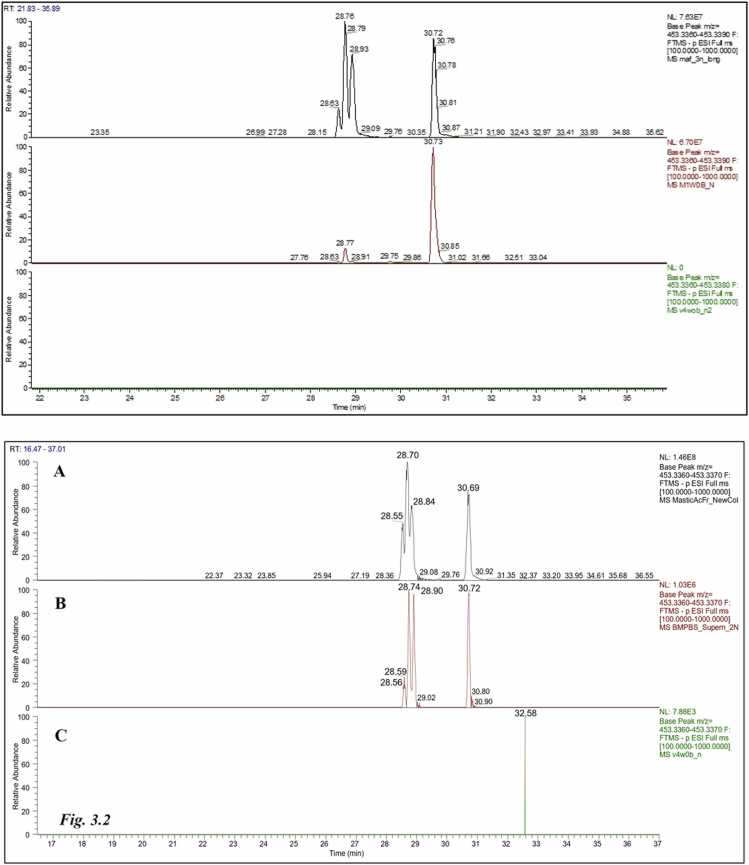
Extracted Ion Chromatograms (XICs) of plasma (Fig. 3.1) and bone marrow extracts (Fig. 3.2) from CMG-treated (B) and untreated (C) rats compared to the respective chromatogram of acidic fraction of CMG (A) at *m/z* 453.3369 in (-) ESI. Moronic acid at Rt= 28.70 min; Oleanonic acid at Rt= 28.84 min; Isomasticadienonic/masticadienonic acids at Rt= 30.72 min in plasma extract.

More specifically, moronic acid, oleanonic acid and isomasticadienonic/masticadienonic acids are isomers with theoretical *m/z* = 453.3370 and molecular formula C_30_H_45_O_3_ in negative mode of detection. Extracted ion chromatogram (XIC) of the acidic fraction of CMG at *m/z* 453.33–453.34 revealed the presence of major features with *m/z* = 453.3370, *m/z* = 453.3368 and *m/z* = 453.3369 at the three major peaks observed in the extract at Rt 28.48 min, 28.65 min and 30.44 min respectively (Fig. 2). In all three cases, the calculated Δmass was < 5 ppm, confirming that the three main peaks of the chromatogram correspond to the three characteristic triterpenic acid isomers (Δmass= 0, 0.44 and 0.22 respectively).

Representative XICs generated from plasma and bone marrow extracts of CMG-treated rats in negative mode of detection at *m/z* 453.33–453.34 are presented in Figures 3.1B and 3.2B respectively. In both cases, the XICs of the treated animals are compared to the XICs of the acidic fraction of CMG (Figures 3.1 A and 3.2 A) and the XICs of the un-treated animals (Figure 3.1 C and 3.2 C).

As shown in Figure 3.2, all three characteristic CMG major peaks, which correspond to the characteristic triterpenic acids, were detected in the XIC of the bone marrow extract from the CMG-treated rats (Rt 28.74, 28.82 and 30.72 min). Calculated Δmass values and isotopic pattern distributions for extracted ions at *m/z* 453.33–453.34 in all CMG-treated animals (Table S1), were all within Δmass < 5 ppm for the compounds of molecular formula C_30_H_45_O_3_, unambiguously confirming the presence of moronic, oleanonic and isomasticadienonic/masticadienonic acids. In contrast, no such features were observed in XIC of plasma from untreated rats at *m/z* 453.33–453.34, confirming the absence of triterpenic acids in the control group.

In plasma extracts from the treated rats, isomasticadienonic/ masticadienonic acids exhibited high bioavailability for CMG-treated rats, whereas moronic acid was less bioavailable (Figure 3.1B). Oleanonic acid was also detected in plasma, albeit at trace levels. Again, no characteristic CMG triterpenic acids were present in the plasma extract of the control animals (Fig. 3.2 C).

MS/MS fragmentation patterns of characteristic CMG triterpenic acids were analyzed studied in detail and also compared to the respective compounds found in the plasma and bone marrow samples. The comparison showed a match to the main and secondary MS/MS fragments of the predominant triterpenic acids (Fig.S1). These findings confirm the unambiguous presence of the characteristic CMG triterpenic acids in the bone marrow and plasma of treated animals [4].

## 4 Discussion

The mammalian *in vivo* micronucleus test is a key assay within genotoxicity test battery typically required internationally for product safety assessment by regulatory agencies [37]. The assay is especially relevant for evaluating genotoxicity hazard, as it can detect cytogenetic damage of a test substance *in vivo*, taking into account factors such as metabolism, pharmacokinetics and DNA repair processes [50]. To our knowledge, no *in vivo* genotoxicity study of Chios Mastic Gum (CMG) has ever been conducted and relevant data are absent from literature. To assess the safety of CMG, a limit test of the mammalian *in vivo* micronucleus was conducted in male Wistar rats which received CMG for 3 consecutive days at 2000 mg/kg bw.

In the micronucleus assay, the proportion of immature erythrocytes (PCEs) among total (immature and mature) erythrocytes or the PCE/ NCE ratio between treated and vehicle-control animals serve as indicator of bone marrow cytotoxicity [37]. Statistical analysis showed that CMG treatment did not significantly change the percentage % of immature erythrocytes and PCE/NCE ratio in CMG-treated rats compared to control rats, suggesting no cytotoxic effect of the treatment on bone marrow erythropoiesis. During the micronucleus test conduct that the percentage of immature erythrocytes in treated animals should remain at least 20 % of that observed in negative control animals when scoring by microscopy [50]. Therefore, the acceptability of a micronucleus test result depends on the degree of cytotoxicity induced by the test material, as a high cytotoxicity can prevent the identification of an increase in the frequency of micronucleated immature erythrocytes due to the inability and/or difficulty to sample an adequate representative population of immature erythrocytes produced following the treatment [39]. In this study the upper acceptable cytoxicity limit, based on the percentage of immature erythrocytes of the negative control group, was 9.33 % (PCE%) (*i.e.* 20 % of the value 46.64 observed in negative control group) of immature erythrocytes in the treated group. This limit was not exceeded indisputably since CMG treatment resulted in approximately 43 % immature erythrocytes in bone marrow.

Apart from cytotoxicity assessment, a depression of the immature to mature erythrocyte ratio can be the main line of evidence showing bone marrow exposure to a test substance [12]. Evidence for bone marrow exposure is especially required when the test chemical did not produce observable toxic effects or target tissue cytotoxicity. It can be obtained from blood analysis in treated animals of the study showing the levels of the test chemical [50]. If blood levels of the testing chemical are adequate, then targeting of bone marrow can reasonably be assumed to occur because bone marrow is a relatively well-perfused organ [8]. CMG treatment reduced marginally the percentage of immature erythrocytes compared to negative control animals, but the reduction was not statistically significant, not providing evidence for bone marrow exposure to CMG. Nevertheless, the presence of CMG in the bone marrow was unambiguously confirmed by the detection of the characteristic CMG triterpenic acids in the extracts of bone marrow and plasma of the treated animals. Bioavailability of CMG compounds 1 h after oral ingestion of 40 mg/kg bw CMG in mice has been shown before for 24Z-isomasticadienonic acid and 24Z-isomasticadienolic acid in plasma by HPLC-MS/MS analysis using reference standards [40].

The primary endpoint of the micronucleus assay of the genotoxicity assessment of the test substance on erythroblasts, resulting from the determination of the frequency of MNPCE in the bone marrow of treated animals compared to negative control. An increase in the frequency of MPCE in test agent-treated animals is an indication of induced chromosome damage [37]. Statistical analysis revealed that CMG treatment did not increase the frequency of MNPCE in bone marrow of CMG-treated rats compared with controls, suggesting a lack of genotoxic effect of CMG on erythroblasts. In contrast, 20 mg/kg cyclophosphamide treatment significantly increased the frequency of MNPCE in the positive control animals compared to negative control, suggesting the good performance of the assay to identify the genotoxic effect of known positive substances. While the absence of a statistically significant increase in the frequency of MNPCE relative to the concurrent negative control is the primary endpoint of the assay, the OECD guideline outlines two more conditions to be concurrently met for a clearly negative result to be concluded. These conditions concern the confirmation of bone marrow exposure to the testing chemical and that all results are inside the distribution of the historical negative control data [50].

Historical control data are essential for validating genotoxicity assays and the interpretation of their results. Historical negative controls are usually measurements representing the spontaneous or background level of genotoxicity in a test system, while positive controls data mainly show the competence of a test system to identify the genotoxic effect of known positive substances. They are both used in comparison with the concurrent control data as part of the assay verification procedure and assay acceptance [22], [50]. Although comprehensive negative control distribution was not established in the present study, the concurrent negative controls aligned well with the range of historical negative control values published in literature. Krishna et al. [38] reported a negative control range of micronuclei in immature erythrocytes to be 1.3–5.3 per 1000 PCE in Wistar rats with a mean of 2.6, based on 360 rats of both sexes for a period of 12 years in their lab. Igl et al. [27] reported that 95 % of the normally distributed historical negative control data analyzed for 480 Wistar HAN rats to have percentages of MNPCE less or equal to 0.28 %.

The statistical design and data analysis framework of the rodent micronucleus assay have undergone multiple revisions over the last decades [1], [24], [25], [33], [34], [50], [52]. Prior to the development of automated scoring methods, the number of scored cells that was recommended by regulatory guidelines was limited at 2000 immature erythrocytes [46] as a result of the tedious microscopic scoring procedure of the assay. Research however showed the lower power of the test when spontaneous micronucleus frequencies are low, i.e less than 0.1 % [25], [35]. The International Workshops on Genotoxicity Testing (IWGT) subsequently recommended that each laboratory should determine the minimum cell sample size required to ensure that the scoring error is maintained below the level of inter-animal variation for spontaneous frequencies [23]. OECD guideline was later revised to recommend the scoring of 4000 immature erythrocytes per animal of at least 5 animal per treatment group [50]. In the present study the number of scored cells was limited to 2000 immature erythrocytes. According to the latest methodological updates, this limitation should be acknowledged; however when spontaneous frequencies reach 0.2 % or higher, scoring of 2000 immature erythrocytes may meet the recommendation made by IWGT [35] for detecting a doubling in the micronucleus frequency with 80 % power at a confidence level of p < 0.05 [23].

To date, *in vivo* genotoxicity studies have evaluated the genotoxicity of CMG in mammals. The present research work is the first *in vivo* study engaged in the assessment of genotoxic activity of CMG by using an assay that examines cytogenetic damage *in vivo* taking into account factors such as metabolism, pharmacokinetics and DNA repair processes. In conclusion, no genotoxicity of CMG was observed at the limit dose of 2000 mg/kg bw on bone marrow erythrocytes. This finding reinforces the safety profile of CMG for potential pharmaceutical and nutraceutical use, consistent with OECD and EMA regulatory framework.

## Author statement

Please see the responses to the reviewers’ comments. We are resubmitting the article including the comments addressed. We remain at your disposal for any further comment of clarification.

## CRediT authorship contribution statement

**Marios Meidanis:** Methodology. **Nikolas Fokialakis:** Conceptualization, Funding acquisition, Project administration, Resources, Supervision, Writing – review & editing. **Kyriaki Machera:** Conceptualization, Investigation, Methodology, Project administration, Resources, Supervision, Visualization, Writing – review & editing. **Eirini-Christina Psarou:** Data curation, Formal analysis, Investigation, Writing – original draft, Methodology, Visualization. **Katerina Kyriakopoulou:** Conceptualization, Data curation, Investigation, Methodology, Supervision, Writing – review & editing, Visualization. **Pelagia Anastasiadou:** Methodology. **Aikaterini Termentzi:** Data curation, Formal analysis, Investigation, Methodology, Supervision, Writing – original draft, Writing – review & editing, Visualization.

## Declaration of Competing Interest

The authors declare the following financial interests/personal relationships which may be considered as potential competing interests: Psarou Eirini-Christina reports financial support was provided by State Scholarships Foundation (MIS-5000432). If there are other authors, they declare that they have no known competing financial interests or personal relationships that could have appeared to influence the work reported in this paper.

## Data Availability

Data will be made available on request.

## Footnotes

Appendix A Supplementary data associated with this article can be found in the online version at doi:10.1016/j.toxrep.2025.102155.
