# Supplementary material for: In vivo genotoxicity of Chios mastic gum in rodent bone marrow micronucleus test
Source: Toxicol Rep. 2025 Oct 31;15:102155. doi: 10.1016/j.toxrep.2025.102155 (PMC12745950; doi:10.1016/j.toxrep.2025.102155)
Supplement: Supplementary file 1 — Supplementary material [file mmc1.docx]

**Supplementary material**

**Table S1:** Features of interest with their chromatographic and spectral characteristics detected in the extracted ion chromatograms of the plasma and bone marrow extracts obtained from three mastic-treated and three untreated rats at *m/z* 453.33-453.34 in negative ESI

| **Animal group** | **Samples**  **no.** | **m/z**  **experim.** | **Rt** | **Intensity** | **Δm** | **Relative isotopic pattern distribution** | | |
| --- | --- | --- | --- | --- | --- | --- | --- | --- |
|  |  |  |  |  |  | **M ion** | **M+1 ion** | **M+2 ion** |
| Mastic-treated rats | 1 | 453.3381 | 28.63 | 509878 | 2.43 | 100 | 31.58 | 3.11 |
|  |  | 453.3379 | 28.77 | 6008115 | 1.99 | 100 | 32.94 | 5.31 |
|  |  | 453.3380 | 30.73 | 26154336 | 2.21 | 100 | 32.60 | 5.38 |
|  | 2 | 453.3378 | 28.60 | 169888 | 1.77 | 100 | 31.74 | - |
|  |  | 453.3378 | 28.76 | 2271773 | 1.77 | 100 | 31.96 | 5.34 |
|  |  | 453.3378 | 30.69 | 22460700 | 1.77 | 100 | 32.77 | 5.68 |
|  | 3 | 453.3379 | 28.61 | 716958 | 1.99 | 100 | 32.80 | 4.50 |
|  |  | 453.3378 | 28.74 | 9728826 | 1.77 | 100 | 32.27 | 5.34 |
|  |  | 453.3378 | 30.70 | 25686210 | 1.77 | 100 | 32.48 | 5.60 |


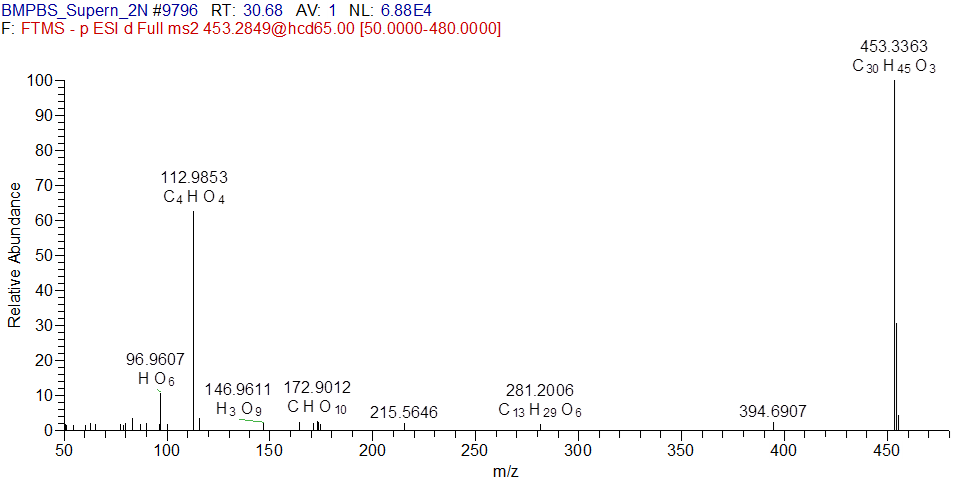


**MS/MS Rt=30.72min**


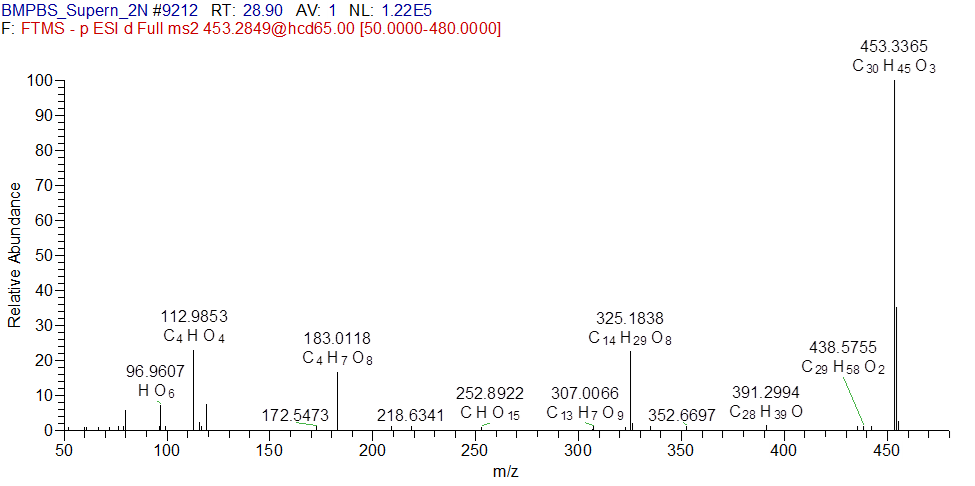


**MS/MS Rt=28.90min**


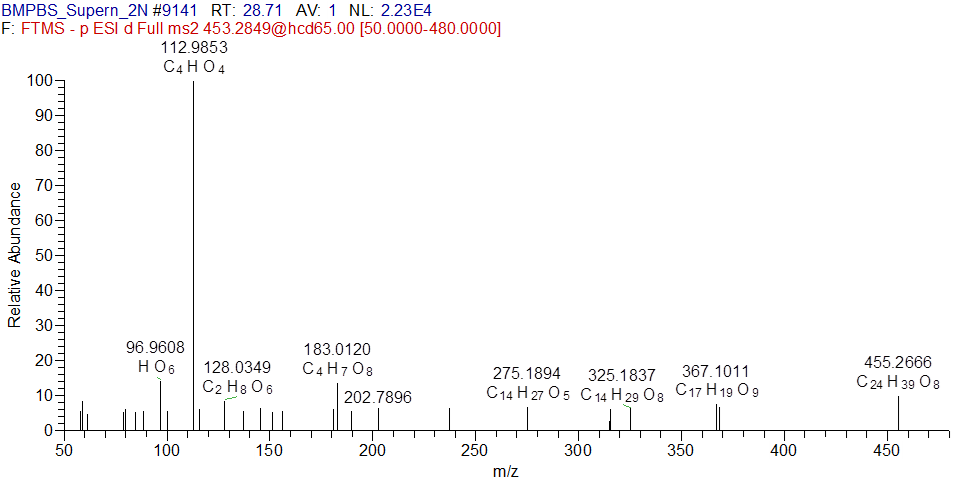


**MS/MS Rt=28.74min**

**Figure S.1**: MS/MS fragmentation spectra of the Bone marrow extracts derived from animals treated with CMG.
